# Reproducibility of Gene Expression Signatures in Diffuse Large B-Cell Lymphoma

**DOI:** 10.3390/cancers14051346

**Published:** 2022-03-05

**Authors:** Jessica Rodrigues Plaça, Arjan Diepstra, Tjitske Los, Matías Mendeville, Annika Seitz, Pieternella J. Lugtenburg, Josée Zijlstra, King Lam, Wilson Araújo da Silva, Bauke Ylstra, Daphne de Jong, Anke van den Berg, Marcel Nijland

**Affiliations:** 1 Department of Pathology and Medical Biology, University Medical Center Groningen, University of Groningen, 9712 Groningen, The Netherlands; jessicaplaca@usp.br (J.R.P.); a.diepstra@umcg.nl (A.D.); a.seitz@umcg.nl (A.S.); a.van.den.berg01@umcg.nl (A.v.d.B.); 2 Center for Cell-Based Therapy, National Institute of Science and Technology in Stem Cell and Cell Therapy (INCT/CNPq), Ribeirão Preto 14051-060, Brazil; wilsonjr@usp.br; 3 Department of Pathology, Cancer Center Amsterdam, Amsterdam UMC, 1105 Amsterdam, The Netherlands; g.t.los@amsterdamumc.nl (T.L.); m.mendeville@amsterdamumc.nl (M.M.); b.ylstra@vumc.nl (B.Y.); d.dejong2@amsterdamumc.nl (D.d.J.); 4 Department of Hematology, Erasmus MC Cancer Institute, University Medical Center, 3015 Rotterdam, The Netherlands; p.lugtenburg@erasmusmc.nl; 5 Department of Hematology, Amsterdam UMC, 1105 Amsterdam, The Netherlands; j.zijlstra@amsterdamumc.nl; 6 Department of Pathology, Erasmus MC, 3015 Rotterdam, The Netherlands; k.lam@erasmusmc.nl; 7 Department of Genetics, Ribeirão Preto Medical School, University of São Paulo, Ribeirão Preto 14049-900, Brazil; 8 Department of Hematology, University Medical Center Groningen, University of Groningen, 9712 Groningen, The Netherlands

**Keywords:** diffuse large B-cell lymphoma, gene expression profiles, reproducibility

## Abstract

**Simple Summary:**

Multiple gene expression signatures with biological or prognostic subgroups have been published in diffuse large B-cell lymphoma (DLBCL). With exception of the cell of origin (COO) classifier, these were not validated in independent cohorts. The aim of the study was to reproduce four gene expression signatures capturing multiple biological subgroups using the NanoString platform. In addition, we aimed to identify potential associations between the signatures and portray the heterogeneity of DLBCL. We show that, in an independent cohort of clinically well-defined patients, these signatures can co-occur in the same patient and that each classifier captures a different aspect of the biological heterogenous panorama of DLBCL. Beside COO, there is clear evidence of different immune and MYC signatures. A direct comparison in our cohort showed that these signatures reflect independent biological features. More comparative studies with gene expression profiles need to be conducted to enable a further integration and to help develop new taxonomy systems for clinical utility.

**Abstract:**

Multiple gene expression profiles have been identified in diffuse large B-cell lymphoma (DLBCL). Besides the cell of origin (COO) classifier, no signatures have been reproduced in independent studies or evaluated for capturing distinct aspects of DLBCL biology. We reproduced 4 signatures in 175 samples of the HOVON-84 trial on a panel of 117 genes using the NanoString platform. The four gene signatures capture the COO, MYC activity, B-cell receptor signaling, oxidative phosphorylation, and immune response. Performance of our classification algorithms were confirmed in the original datasets. We were able to validate three of the four GEP signatures. The COO algorithm resulted in 94 (54%) germinal center B-cell (GCB) type, 58 (33%) activated B-cell (ABC) type, and 23 (13%) unclassified cases. The MYC-classifier revealed 77 cases with a high MYC-activity score (44%) and this MYC-high signature was observed more frequently in ABC as compared to GCB DLBCL (68% vs. 32%, *p* < 0.00001). The host response (HR) signature of the consensus clustering was present in 55 (31%) patients, while the B-cell receptor signaling, and oxidative phosphorylation clusters could not be reproduced. The overlap of COO, consensus cluster and MYC activity score differentiated six gene expression clusters: GCB/MYC-high (12%), GCB/HR (16%), GCB/non-HR (27%), COO-Unclassified (13%), ABC/MYC-high (25%), and ABC/MYC-low (7%). In conclusion, the three validated signatures identify distinct subgroups based on different aspects of DLBCL biology, emphasizing that each classifier captures distinct molecular profiles.

## 1. Introduction

Diffuse large B-cell lymphoma not otherwise specified (DLBCL NOS) is a heterogeneous disease that accounts for 40% of all mature B-cell neoplasms [1]. While the outcome of patients with a low-risk disease as determined by the clinical International Prognostic Index (IPI) score is excellent, the prognosis of patients with high-risk DLBCL remains dismal with 40% of patients failing first-line treatment with rituximab, cyclophosphamide, doxorubicin, vincristine, and prednisolone (R-CHOP) [2].

The cell-of-origin (COO) concept was first published in 2000, dividing DLBCL based on gene expression profiles (GEP) in germinal center B-cell (GCB) type, activated B-cell (ABC) type, and unclassified cases [3]. The COO subgroups were shown to have distinct features indicating involvement of different oncogenic pathways. Patients with ABC-type DLBCL showed an inferior outcome in a retrospective setting [3]. So far, clinical studies targeted towards specific oncogenetic characteristics of ABC-type DLBCL patients, e.g., combining the small molecules bortezomib, ibrutinib, and lenalidomide to R-CHOP have not been successful to improve outcome, which underpins that a simple dichotomy to define DLBCL does not sufficiently capture the oncogenetic complexity of this disease [4,5,6]. Moreover, about 15% of DLBCL cases remain unclassified and these cases do not have other characteristic aberrations that can advise the treatment of these patients [3].

In 2017, the World Health Organization (WHO) classification categorized high-grade B-cell lymphomas with an *MYC* rearrangement combined with a *BCL2* and/or *BCL6* rearrangements separately as double hit high-grade B-cell lymphoma (HGBCL DH) and DLBCL cases with high-grade morphology that lack these concurrent hits as HGBCL NOS [1]. The poor outcome in HGBCL patients justified a dedicated treatment approach within this group, those cases with *MYC* immunoglobulin heavy or light chain gene (*MYC-IG*) rearrangements were especially shown to have an inferior prognosis [7]. Despite the use of more intensive chemotherapy and small molecules for patients with HGBCL DH, randomized clinical trials are lacking [8,9].

From 2010 onward, the focus in unraveling the biology has been on deciphering the mutational landscape of DLBCL using next generation sequencing (NGS) approaches [10,11,12,13,14]. The subgrouping as based on the observed mutational profiles showed enrichment of specific mutations for either ABC or GCB subsets. Although the mutational patterns are not mutually exclusive, they were at least partially correlated to COO, but much less to the presence of *MYC* rearrangements [10,11,12,13,14].

In parallel to the mutational landscape studies, multiple GEP studies have identified distinct biological DLBCL subgroups [15,16,17,18,19,20,21,22,23,24,25,26,27,28,29]. These studies have generated profiles related to tumor cell characteristics including MYC activity and the micro-environment composition. However, the biological relevance and clinical impact of these gene signatures have not resulted into incorporation in clinical trials. Around the start of this study, twelve different GEP classifiers had been published [15,16,17,18,19,20,21,22,23,24,25,26].

The aim of this study was to reproduce four biology driven gene expression signatures in a large cohort of clinically well annotated DLBCL NOS/HGBCL samples from the HOVON-84 trial using the NanoString platform, which permits robust amplification free GEP analysis of RNA from formalin-fixed paraffin-embedded tissue with minimal background signal [30]. Selection of the four gene expression signatures was based on the biological features presenting COO, MYC activity, oxidative phosphorylation (OxPhos), B-cell receptor (BCR) signaling, and the micro-environment as well as the potential reproducibility of the classifiers (FFPE based, number of genes, and availability of algorithms) [15,16,17,18]. In addition, we studied whether the reproducible gene expression profiles are independent of each other and whether their combined use can indicate distinct DLBCL NOS/HGBCL subgroups. Finally, we tested potential associations with clinical features in a well-defined population of patients with DLBCL NOS/HGBCL.

## 2. Materials and Methods

### 2.1. Patient Cohort

HOVON-84 is a multicentric, randomized phase III trial, with no benefit of the intensification of rituximab combined with 2-weekly CHOP chemotherapy in patients with newly diagnosed DLBCL. At the time of the study HGBCL DH was not considered a distinct entity and as such was included in the trial [31]. The study was conducted in accordance with the ethical guidelines mandated by the Declaration of Helsinki and approved by all relevant institutional review boards or ethical committees. Written informed consent, including use of biopsy material for research purposes, was obtained from all patients. The HOVON-84 trial included 574 patients and good quality NanoString (Seattle, WA, USA) data could be generated for 175 patients. This cohort forms the core of the present study. In the other 399/574 patients, no representative formalin-fixed paraffin embedded (FFPE) biopsy material was available for this study (blocks not available for study, blocks exhausted, or insufficient quality) or NanoString data were of insufficient quality.

Clinical characteristics of the 175 HOVON-84 patients studied in this report as well as the characteristics of the total cohort and the original GEP signatures cohorts are listed in Table S1. No statistically significant differences were observed between the cases included in the present study and the entire HOVON-84 cohort, making the samples used in this study a representation of the entire cohort. (Gender *p*-value *=* 0.5; Age *p*-value *=* 0.5; Stage *p*-value *=* 0.3; LDH levels *p*-value *=* 0.1; aaIPI *p*-value *=* 0.2; OS *p*-value *=* 0.06; COO *p*-value *=* 0.9.)

### 2.2. Immunohistochemistry

Immunohistochemistry (IHC) was performed as part of previous studies by the Lunenburg Lymphoma Biomarker Consortium (North Bethesda, MD, USA) [7,32] and available for 167 DLBCL patients for CD10, MUM1, and BCL6. In addition, BCL2 and MYC IHC was performed for 161 DLBCL patients using routine diagnostic procedures on tissue microarrays. Scoring of CD10, MUM1, and BCL6 staining and subsequent classification as GCB or non-GCB was performed according to the Hans algorithm [33]. MYC IHC was scored as the percentage of positive tumor cells as estimated by an experienced hematopathologist in 10% increments. Lymphomas were defined as double expressors (DE) based on MYC positivity in ≥40% and BCL2 positivity in ≥50% of the tumor cells [34]. For correlation to the MYC gene signature as published by Carey et al. [16], we used a cutoff of ≥50% positive tumor cells for MYC-High and <50% for MYC-Low consistent with the cutoff as defined in this paper. For MHC-II (HLA-II), IHC was performed on tissue microarrays and cores were scored for intensity of staining. No or weak staining was classified as MHC-II low and all other cases were classified as MHC-II high [35].

### 2.3. Detection of Chromosomal Translocations in BCL2, BCL6, and MYC

Fluorescence in situ hybridization (FISH) for *MYC, BCL2,* and *BCL6* was performed on 152, 148, and 153 cases, respectively, with break apart probes from Vysis LSI, Abbott (Chicago, IL, USA). Scoring was performed as described previously [7,32].

In addition to the FISH, targeted NGS was performed for 140 samples to identify structural variants (SV) in *MYC*, *BCL2*, and *BCL6* using the protocols as previously described [36]. The SV information was combined with the FISH results to classify cases as HGBCL DH, regarding all cases with a positive result for either FISH (8) or NGS (7) or both (125) as positive.

### 2.4. Gene Expression Profiling

For a total of 175 samples, we were able to obtain sufficient good quality RNA with FFPE RNeasy Kit (Qiagen, Hilden, Germany) for analysis on the NanoString Platform. The core set of probes for 117 genes (see Supplementary Table S2 for a complete overview) was hybridized to 100–200 ng of RNA for 16 h at 65 °C. Samples were loaded on an nCounter SPRINT Cartridge and processed on the nCounter SPRINT™ Profiler. The expression data were analyzed using Nanostring’s nSolver analysis software (version 3.0). Registered counts passing the standard QC parameters were used for further analysis. The normalized data were scaled and transformed to log2.

### 2.5. COO Classifier

For COO classification, raw counts obtained by NanoString gene expression analysis for all genes of the algorithm were uploaded at the Lymphoma/Leukemia Molecular Profiling Project (LLMPP) website (https://llmpp.nih.gov/LSO/LYMPHCX/lymphcx_predict.cgi, accessed on 12 September 2017) to run the Lymph2Cx classifier [15].

### 2.6. MYC Activity Score

To reproduce the MYC activity score we used the selection and bioinformatics strategy as reported by Carey et al. [16], since the algorithm is not publicly available. In brief, we used their original training cohort as training set of the elastic net classifier. This training set included 14 cases scored as MYC-low based on positive staining in <40% of the tumor cells and 16 cases as MYC-high based on positive staining in >60% of the tumor cells. The classifier was subsequently applied to the HOVON-84 (*n* = 175) test set.

The training dataset was normalized with the R package NanoStringNorm [37], considering the sum of the expression values to estimate the technical assay variation, the mean to estimate background count levels, and the sum of the six housekeeping genes to normalize for the RNA sample content. In addition, the data were log2 transformed. The alfa and gamma parameters were set at 0.1 and the classification accuracy was assessed with the Leave One Out Cross Validation (LOOCV), as in the original publication. A cutoff of 0.5 was used to stratify the tumors with high and low MYC activity score.

The importance of each gene was calculated based on combinations of the absolute values of the weights as reported by Gevrey et al. [38]. All the analyses were conducted with the R package caret [39]. The spearman’s correlation was used to evaluate the association between the MYC activity score and MYC IHC values and the predictions were compared with the outcome of the IHC staining.

### 2.7. Monti Consensus Clustering

Briefly, the three consensus clustering approaches applied were Hierarchical Clustering (HC) considering the Euclidean distance, Self-Organizing Maps (SOM) with the R packages ConsensusClusterPlus [40] and Kohonen [41], and the Gaussian Finite Mixture Models algorithm (which represents the probabilistic clustering (PC)) using the R package mclust [42]. To define the best number of clusters, we used 80% of resampling on 200 replicates for each clustering algorithm, as in the original paper. Consensus matrices including two to nine clusters were built and evaluated by the relative change in area under CDF curves or Bayesian Information Criterion (BIC) metrics. Confusion matrices were used to determine the number of samples assigned to similar clusters by any 2 algorithms.

HOVON-84 samples with the same classification by all three algorithms (“meta-consensus”) were defined as samples belonging to the main clusters. For the remaining HOVON-84 samples, we built a naive-Bayes classifier with the R package caret [39]. The naïve-Bayes classifier was first trained with the samples from the original meta-consensus clustering study and subsequently used to predict the cluster membership for the remaining HOVON-84 samples, similar to the approach applied in the original publication.

### 2.8. Immune Ratio

To reproduce the prognostic marker based on the expression ratio between immune effectors and inhibitory (immune checkpoint) genes, we followed the approach as published by Keane et al. [18]. We decided to focus on their main finding, which was the prognostic significance of the *CD4* × *CD8* to *CD163*:*CD68* × *PD-L1* ratio. This immune ratio was additive and independent to the revised-IPI and COO in the original paper. The ratio was calculated using the log2 scaled gene expression values and to assess the prognostic value of this ratio in the HOVON-84 cohort we used the Keane proposed cut-off (−0.278958829) to stratify samples into high and low expression ratio subgroups.

### 2.9. Statistical Analysis

To compare categorical data, we used Fisher’s Exact Test or the X^2^ test, where applicable. The Kaplan–Meier method was used to estimate the overall survival (OS) and progression free survival (PFS). Univariable and multivariable Cox proportional hazard regression models and Wald *p*-values were used to evaluate the prognostic impact and statistical significance. All the analyses were performed in R 3·6·2 [43]. We did not separately analyze patients per study treatment arm since PFS and OS were similar, and treatment regimens differed on Rituximab-dose only. Patients with significant therapy protocol violations were not included.

## 3. Results

### 3.1. Study Design

In addition to the widely used COO signature to classify DLBCL cases (Scott et al. 2014 [15]), we prioritized three additional signatures that were NanoString based, since it is a reproducible technology by different laboratories, available at that time, and reflected different biological aspects. The three selected signatures included MYC activity score (Carey et al. 2015 [16]), Monti consensus clustering (Monti et al. 2005 [17]), and the immune-ratio signature (Keane et al. 2015 [18]). As the COO classifier and immune ratio classifiers were both based on a limited number of genes, we included all genes and applied the published algorithms. For the two much larger classifiers, i.e., MYC activity score and the consensus clustering, with algorithms that had to be re-designed, we followed a different approach. We first recreated the clustering and/or classification algorithms and tested their performance on the originally reported cohorts, with the original set of genes. To make a subsequent clinical application feasible, we reduced the gene list, by prioritizing the genes with the strongest contributions to the algorithms and applied the validated algorithms on the original cohorts to establish the effectivity of our selected gene set.

### 3.2. Performance of the MYC Activity Score Using a Subset of the Genes

The MYC activity score algorithm was first reproduced in the Carey cohort using the original set of 61 genes (Figure S1A). Next, we tested the validated algorithm on our subset consisting of 34 genes (Figure S1B). Although the impact of the genes in the classifier was different from the original publication for both gene sets [16], *MYC* had the highest impact consistent with the original paper. We observed a good correlation between the MYC activity score and the percentage of tumor cells staining positive for MYC in the Carey training set cases using both the initial gene set and the subset included in our analysis (Figure S1C,D). Moreover, we observed a perfect match of the MYC activity score with the MYC expression as determined by IHC in the training set (Table S4).

### 3.3. Performance of the Monti Consensus Clustering Algorithm Using a Subset of the Genes

We first reproduced the Monti consensus clustering into Oxidative phosphorylation (OxPhos), B-cell Receptor/Proliferation (BCR), and Host response (HR) groups using the dataset of Monti et al. [17]. The three algorithms revealed three subgroups consistent with the original Monti publication using 1112 annotated genes from the 2118 microarray probes. Meta-consensus clustering revealed an initial classification of 115 out of 176 samples (Figure S2A). This showed that our algorithm correctly recapitulates the original clustering patterns as reported by Monti et al. [17].

After successful reproduction of the original clustering pattern, we evaluated the performance of the algorithm on our selected subset of genes, which included probes ranking in the top 50 most relevant probes to define each of the three biologic clusters, as specified by Monti. In total, this gene set comprised 47 out of 1112 annotated genes (12 out of 342 OxPhos related genes, 14 out of 344 BCR/Proliferation related genes, and 21 out of 427 HR related genes). This revealed for 130 of the 176 samples of the Monti cohort a consistent clustering, indicating that our selection of genes correctly assigned the majority of the samples to the three clusters (Figure S2B).

### 3.4. COO Classifier in HOVON-84

**Figure 1:**
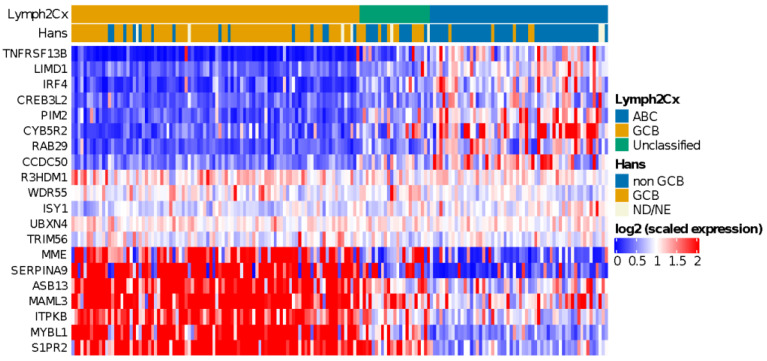
Heatmap showing relative expression levels of the COO genes used to classify cases using the Lymph2Cx algorithm. A clearly distinct gene expression pattern can be observed for ABC and GCB subtype DLBCL cases.

Application of the COO classifier revealed 94 (54%) GCB, 58 (33%) ABC, and 23 (13%) unclassified cases (Figure 1). According to Hans classification, 91 cases (54%) were classified as GCB and 76 (46%) as non-GCB. We observed a significant association (*p* < 0.00001) between the COO classifier and the Hans algorithm (Table S3). Sensitivity and specificity values were 91% and 84% for ABC and non-GCB comparison and 84% and 91% for GCB classes, as previous reported [44]. We also found that ABC cases were enriched in older patients (>60 years) (*p* < 0.002), as reported by Klapper et al. in 2012 [45].

### 3.5. MYC Activity Score in HOVON-84

**Figure 2:**
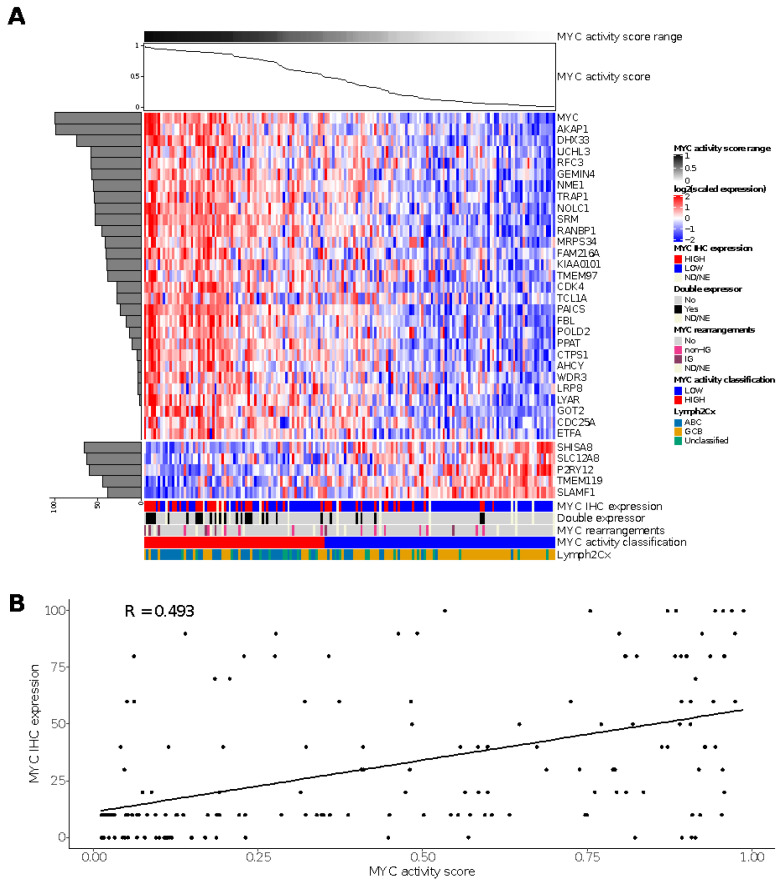
Results of the MYC activity classifier in the HOVON-84 cohort: (**A**) Heatmap for relative expression of the profiling panel including the relative contribution of each gene to the classifier (horizontal, shaded bar graph on the left) and the MYC activity score for the HOVON-84 cohort (line graph on top of the figure). (**B**) Spearman’s correlation between MYC activity score and MYC IHC expression for the 161 samples of the HOVON-84 cohort. ND, Not Done; NE, Not Evaluable.

For the HOVON-84 cohort, we classified 77 cases (44%) as MYC high and 98 (56%) cases as MYC low (Figure 2A). The sensitivity and specificity values relative to the MYC IHC score based on staining in at least 50% of the tumor cells were 0.65 and 0.65, respectively. The negative and positive predictive values were 0.82 and 0.43, respectively, for the identification of MYC IHC expression (Table S4). A significant correlation (R 0.493; Fisher exact test *p* = 0.006) was observed for the MYC activity score and the percentage of tumor cells staining positive for MYC in the HOVON-84 cohort (Figure 2B).

The high-activity MYC group was enriched for DE (*p* < 0.00001) and ABC-type (*p* < 0.00001) lymphoma. There was no association between the MYC activity score and HGBCL DH. Thus, the MYC activity score could be validated in the HOVON-84 cohort and showed a clear correlation with DE and ABC-type lymphomas.

### 3.6. Monti Consensus Clustering in HOVON-84

For the HOVON-84 cases, application of the validated algorithms revealed two as the most optimal number of clusters (Figure S3A,B). The meta-consensus clustering exhibited a consistent subgroup for all three algorithms for 67 (38%) HOVON-84 cases. These cases were characterized by two profiles: a larger cluster (43 samples–24%) with high expression of both BCR/proliferation and Oxphos genes (BCR/Proliferation/Oxphos-high cases) and a cluster (24 samples–14%) characterized by a high expression of HR genes (HR-high cases). Thus, in contrast to the findings of Monti [17], the non-HR cases were not characterized by a differential expression of BCR/proliferation and Oxphos genes.

**Figure 3:**
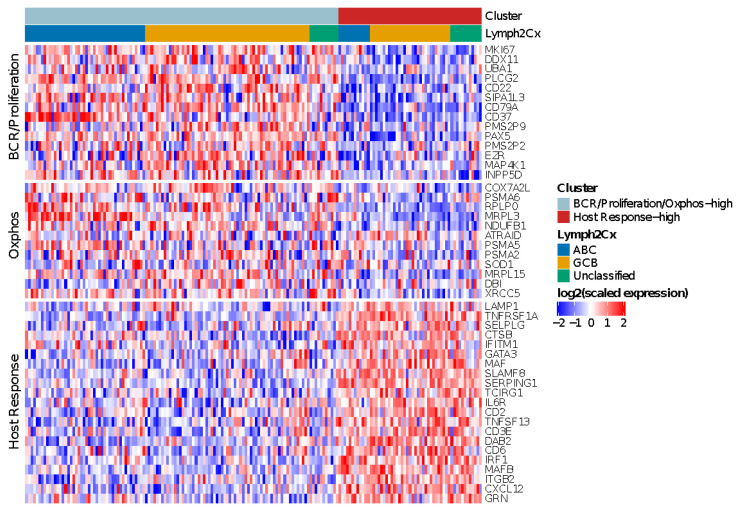
Heatmap showing the relative expression levels of BCR/Proliferation, Host Response (HR) and Oxphos genes used to reproduce the Monti consensus clustering. The HR cluster was validated in 55/175 HOVON-84 cases; the remaining cases showed low expression of HR genes, but no distinct clustering based on BCR/Proliferation and Oxphos genes.

Next, we followed the same strategy as reported by Monti [18] to define the most likely cluster for the remaining 108 (62%) HOVON-84 cases. This revealed a consensus BCR/proliferation/Oxphos-high cluster signature for 77 (44%) samples and a consensus HR cluster-signature for 31 (18%) samples. In total 120 (77 + 43) cases were classified as BCR/proliferation/OxPhos-high and 55 (24 + 31) cases (31%) as HR cluster (Figure 3).

The clusters were distributed across all three COO groups, with an enrichment of BCR/proliferation/Oxphos-high cluster in ABC cases (*p* = 0.02). In summary, the HR cluster, but not the BCR/Proliferation and Oxphos clusters could be validated in the HOVON-84 cohort.

### 3.7. Immune-Ratio Classifier

The immune ratio [18] revealed a ratio under the cut-off for 74 (42%) of the HOVON-84 samples (Figure S4).

### 3.8. Comparison of the Reproduced GEPs

**Figure 4:**
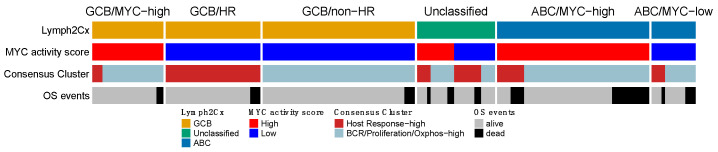
Overlap of the gene expression signatures that were validated in the HOVON-84 cohort. The three signatures show no clear overlap and together are likely to capture different aspects of DLBCL biology. OS events were observed in each of the six clusters, with a slight enrichment in the ABC/MYC-high group.

Next, we compared the four expression signatures to establish a potential overlapping or shared biology. We focused on the overlap among the three larger GEP profiles and separately analyzed a potential overlap with the immune-ratio signature. The mutual impact of the COO, MYC, and the HR group of the Monti consensus clustering signatures, is shown in Figure 4. The overall picture indicated that the three profiles reflect different aspects of lymphoma biology, with no clear overlap. Most ABC cases were characterized by high MYC activity (45/58–77.6%; *p* < 0.00001), whereas the consensus HR-cluster was uncommon (12/58–20% samples) and showed no clear pattern in relation to the MYC signature (*p* = 0.44). The GCB samples largely consisted of MYC-low activity cases (73/94–77.7%; *p* < 0.00001), with in about one third of the cases a consensus HR-cluster (31/94–33%). The smaller GCB/MYC-high group was enriched for DH (*p* = 0.019), DE (*p* = 0.41) and MYC immune positive cases (*p* = 0.002). About half of the cases in the COO-Unclassified cases were high MYC activity (11/23–48%) and consensus HR cases (12/23–52%). Thus, the MYC and consensus clustering profiles within the COO-Unclassified cases showed an intermediate profile and did not indicate a closer association with either ABC or GCB-type DLBCL.

The high immune-ratio subgroup was associated with the HR consensus cluster (OR = 2.82; *p* = 0.003) and with the high MYC activity cluster (OR = 0.387; *p* = 0.003) while no association was found with the COO classifier (Figure S5). We evaluated the correlation of MHC-II IHC with the different gene signatures as proposed by Ennishi et al. [45]. We did not identify an association of MHC-II-IHC high and HLA-II-IHC with COO, MYC activity score, Monti consensus clustering, and immune-ratio signatures.

### 3.9. Prognostic Impact of Validated Signatures

**Figure 5:**
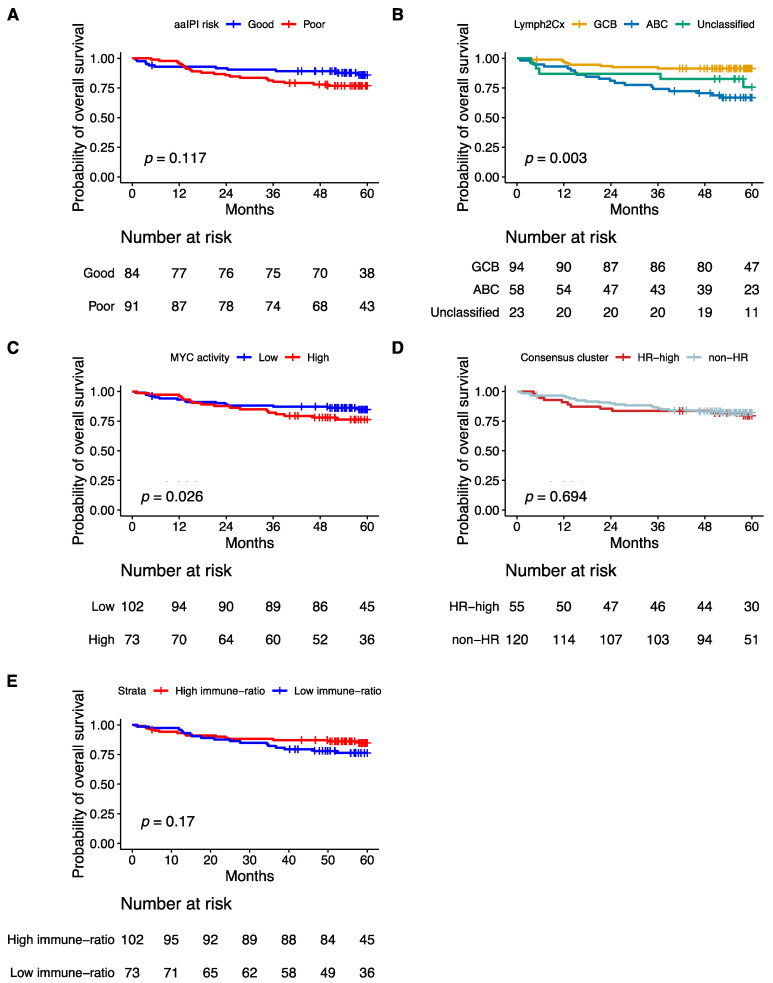
Kaplan–Meier curves showing overall survival of 175 patients from the HOVON-84 cohort: According to (**A**) the aaIPI, (**B**) the COO classification defined by the Lymph2Cx algorithm, (**C**) the Monti consensus clusters, (**D**) the MYC activity classifier, (**E**) the immune-ratio subgroups.

**Figure 6:**
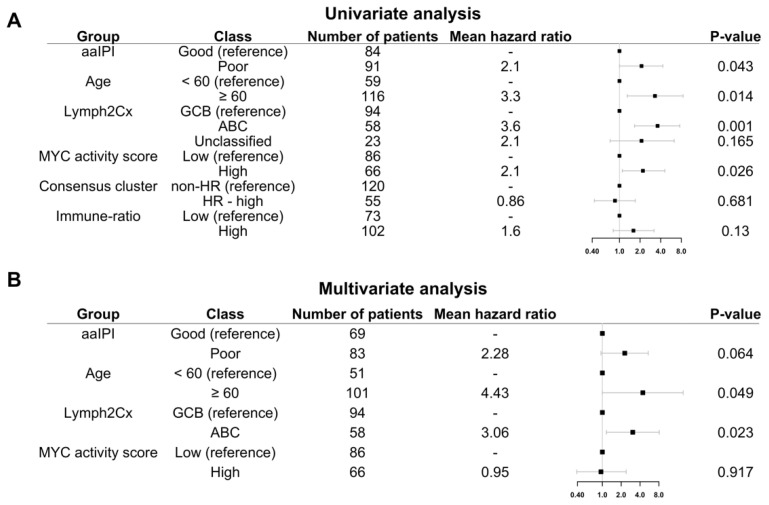
Five-year OS of HOVON-84 patients: (**A**) Forest plot with the univariate effect of the clinical variables and GEP signatures. (**B**) Forest plot with the multivariate effect of clinical variables and GEP signatures. In this cohort, only the COO as defined by the Lymph2Cx remains significant.

Consistent with previous publications, poor aaIPI, which does not consider age, advanced age (>60 years), the COO ABC-type, and the high MYC activity score were significantly associated with poor five-years OS in a univariate analysis (Figure 5A–C and Figure 6A). The Hans non-GCB subgroup was associated with poor survival in HOVON-84 samples (*p =* 0.01) (Figure S6A). The HR cluster of the Monti consensus clustering had no impact on survival consistent with the original report (Figure 5D). In contrast to the original paper, we could not validate the prognostic relevance of the immune-ratio classifier (Hazard ratio 1.6; *p* = 0.2) (Figure 5E). Other MYC molecular features known to impact patient’s survival based on the literature such as high MYC IHC expression and DE and DH events had no impact on five-years OS (Figure S6B–D).

Multivariate analysis including the four variables significant in the univariate analysis, i.e., aaIPI, age, GCB versus ABC, and MYC activity score showed that only the COO ABC-type remained prognostic (Hazard ratio 3.06; *p =* 0.023) (Figure 6B).

## 4. Discussion

In this NanoString-based GEP-profiling study we used a selected set of genes to validate previously published signatures. With this limited set of genes, we were able to faithfully reproduce the classifications of the original MYC activity score and Monti consensus clustering algorithms. Besides the COO, we also reproduced the HR cluster of the Monti consensus clustering and the MYC activity signature in the well-defined HOVON-84 study population. We were not able to reproduce the BCR or Oxphos signatures of the Monti consensus clustering. In contrast to the original study, we did not observe a significant difference in survival for the immune-response ratio [18].

Although COO is well established, its prognostic value has been disputed [46]. The poor survival as observed for ABC type DLBCL has been used as a starting point for the design of several clinical trials. Combination of lenalidomide and ibrutinib in relapsed DLBCL showed efficacy particularly in patients with Hans-based non-GCB type DLBCL, supporting clinical relevance of the COO concept [47]. Molecular subclassification with or without considering the COO might be used for the design of more focused clinical trials to improve the outcome of specific DLBCL subgroups [12,13,48].

There are several ways to categorize DLBCL cases based on MYC status: i.e., DH (FISH), DE (IHC), and GEP classifiers. The more recently published DH GEP signature enables identification of cases with cryptic *MYC* rearrangements [28,49]. The biological rationale underlying the MYC activity GEP signatures is evident, since this enables capturing of the indirect activity of MYC; although, implementation of such a profile in clinical practice warrants further development. We were able to reproduce the Carey [16] MYC classifier, but the impact of the genes was different from the original paper. A possible explanation for this difference might be that our cohort includes cases with the entire spectrum of percentage positive cells, whereas the training set from Carey has been selected for cases with more extreme IHC-based MYC scores. The differences in the spectrum of MYC scores possibly explains the weaker correlation and lower positive predictive value for identifying HGBCL-DH in the HOVON-84 samples. The high MYC activity group showed a poor outcome, while DH cases had no impact on OS, probably because we observed a limited number of events in the HOVON-84 cohort of patients with a HGBCL-DH. Nevertheless, comparison of different algorithms is needed to select the best algorithm for clinical application.

The HR cluster was the most eminent profile identified by Monti et al. [17] and highlights the tumor microenvironment as a defining feature. HR cases had increased expression of genes associated with T-cell-mediated immune responses, the classical complement pathway, coregulated inflammatory mediators, and connective tissue components. A micro-environment-based GE profiling described by Lenz et al. [20] (macrophage 1 (M1) and macrophage 2 signatures (M2)) showed a clear distribution among the COO subgroups. Although we did not include the genes to validate the M1 and M2 signatures, the inflammatory response described by Monti et al. [17] is characterized by and recapitulates this profile. More recently, a novel interest for micro-environment-based GEP using CIBERSORT [50] or single-cell RNA-seq analysis has arisen. The clinical value of the HR signature might become more important with the rise of a whole new range of therapies, including chimeric antigen receptor T cells (CAR T-cells) and bispecific monoclonal antibodies, where the nature of the micro-environment is likely to be important for a durable clinical response [51].

Recently, three additional signatures associated with *MYC* and tumor microenvironment were reported [27,28,29]. However, the limited overlap with our gene panel precluded validation of these signatures. Most likely, part of these studies basically looks at similar underlying biology, including two different *MYC* classifiers but using a different set of genes [16,28]. There was no evident overlap among the COO, HR, and MYC activity subgroups, emphasizing that each classifier captures a different aspect of the biological heterogeneous panorama. In a recent review, a reclassification of DLBCL based on molecular genetics and gene expression profiling was proposed by Ennishi et al. [48]. We now show experimental evidence supporting their proposed subgrouping, with GCB-type DLBCL samples being split in three subgroups as high MYC activity non-HR cases and low MYC activity score splitting in either HR or non-HR cases. Similarly, ABC DLBCL were mainly characterized by high MYC activity scores. However, we did not find any associations with MHC-II expression in the HOVOV-84 cohort. So, our data mostly support the newly proposed classification by Ennishi et al. and emphasizes the importance of biology-driven molecular subgroups.

Profiling of a dedicated subset of genes has become feasible using the Nanostring gene expression system even on FFPE tissue samples containing poor quality RNA. We show a reliable classification of DLBCL cases with multiple gene expression signatures even when using a limited set of genes. Further validation studies are required to link the established signatures to more recently published GEP and mutational signatures and elucidate the complete spectrum of the very heterogenous group of DLBCL. Beyond the biological relevance, COO and MYC activity gene expression signatures had an impact on survival. This highlights the potential of combining different classifiers to improve the identification of high-risk cases and emphasizes the need to integrate these signatures in future clinical trials. The limited gene set required to generate the signatures in combination with the freely available algorithms enables a strait forward and cost-effective implementation. Moreover, combining multiple GEP may lead to improved stratification of patients into specific molecular subgroups that may be sensitive to specific targeted therapeutics.

## 5. Conclusions

In conclusion, we showed that COO, MYC activity score, and the HR cluster of the Monti consensus clustering were reproduced in the HOVON-84 cohort. These three signatures identify distinct subgroups based on different aspects of DLBCL biology, emphasizing that each classifier captures distinct molecular profiles, offering a framework for clinical trials.

## Data Availability

Gene expression data are available under request. The clustering algorithms are available at: https://github.com/jessicaplaca/HOVON84 (accessed on 27 December 2021).

## Supplementary Materials

The following are available online at https://www.mdpi.com/article/10.3390/cancers14051346/s1, Figure S1: MYC activity classifier for the original Carey training cohort, Figure S2: Consensus clusters in original Monti cohort, Figure S3: Identification of consensus clusters in the HOVON-84 cohort using 47 selected genes following the approach as published by Monti, Figure S4: Reproduction of the immune ratio. Distribution of the CD4*CD8:(CD163:CD68)*PD-L1 immuno-ratio for HOVON-84 cohort, Figure S5: Overlap of Immune ratio, COO classifier, and Consensus Cluster signatures in the HOVON-84 cohort, Figure S6: Kaplan–Meier curves for overall survival of the HOVON-84 cohort for (a) the COO classification defined by Hans; Table S1: Overview of characteristics of the HOVON-84 and previously published cohorts, Table S2: List of genes used to classify the four GEP using quantification by the Nanostring platform, Table S3: Comparison of cell of origin (COO) allocation between COO classifier and Hans algorithm, Table S4: Performance of MYC activity classifier in the Carey training and HOVON-84 test sets.
