# Supplementary material for: Reproducibility of Gene Expression Signatures in Diffuse Large B-Cell Lymphoma
Source: Cancers (Basel). 2022 Mar 5;14(5):1346. doi: 10.3390/cancers14051346 (PMC8909016; doi:10.3390/cancers14051346)
Supplement: Supplementary file 1 [file cancers-14-01346-s001.zip › cancers-1553472-supplementary.pdf]

Supplementary Materials: Reproducibility of gene expression signatures in diffuse large B-cell lymphoma

Jessica Rodrigues Plaça, Arjan Diepstra, Tjitske Los, Matías Mendeville, Annika Seitz, Pieternella J. Lugtenburg, Josée Zijlstra, King Lam, Wilson Araújo da Silva Jr, Bauke Ylstra, Daphne de Jong, Anke van den Berg and Marcel Nijland

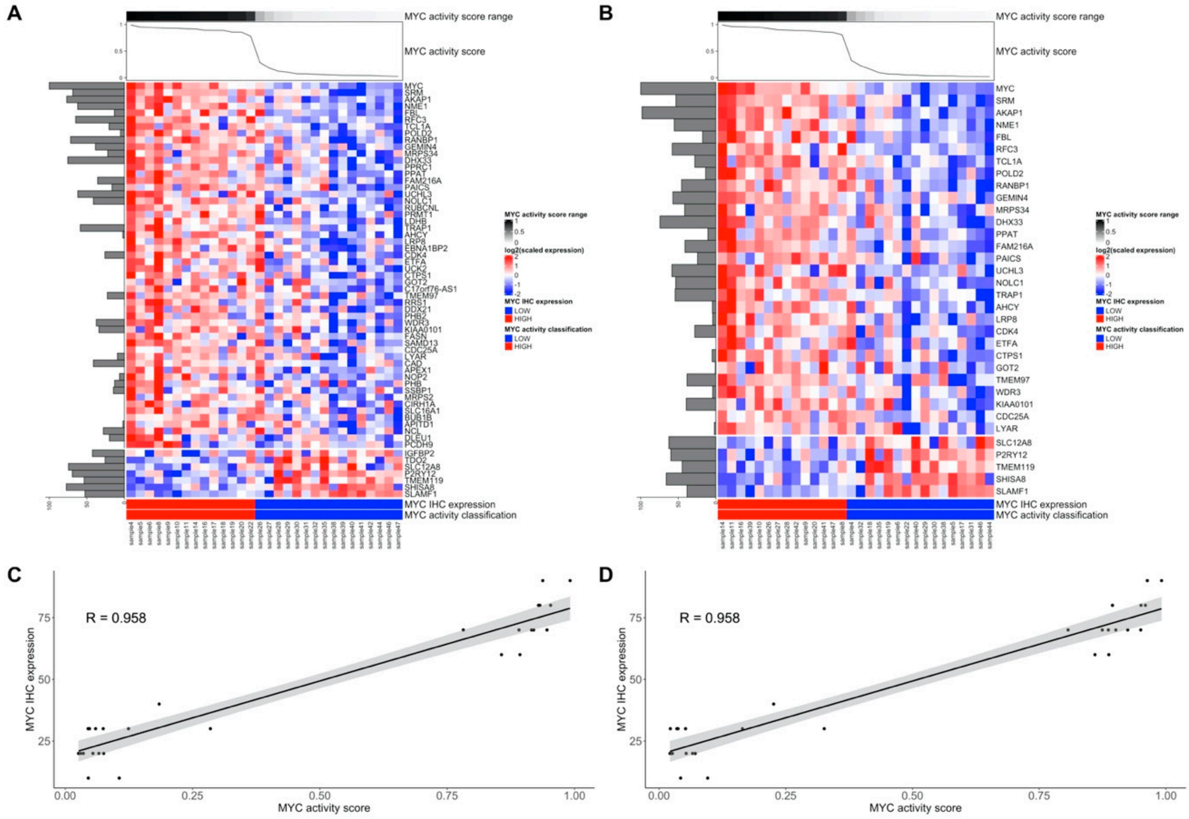

Figure S1. MYC activity classifier for the original Carey training cohort. (**a**) Heatmap with relative expression levels of the 61 genes including the relative contribution of each gene to the classifier (horizontal, shaded bar graph) and the MYC activity score (line graph). (**b**) Heatmap with relative expression levels of the 27 genes selected for our study including the relative contribution of each gene to the classifier (horizontal, shaded bar graph) and the MYC activity score (line graph). (**c**) Spearman's correlation between MYC activity score and MYC IHC expression for the 30 samples of the Carey training cohort considering 61 genes in the model. (**d**) Spearman's correlation between MYC activity score and MYC IHC expression for the 30 samples of the Carey training cohort considering 45 genes in the model. The selected set of 45 genes recapitulates the original MYC activity clusters.

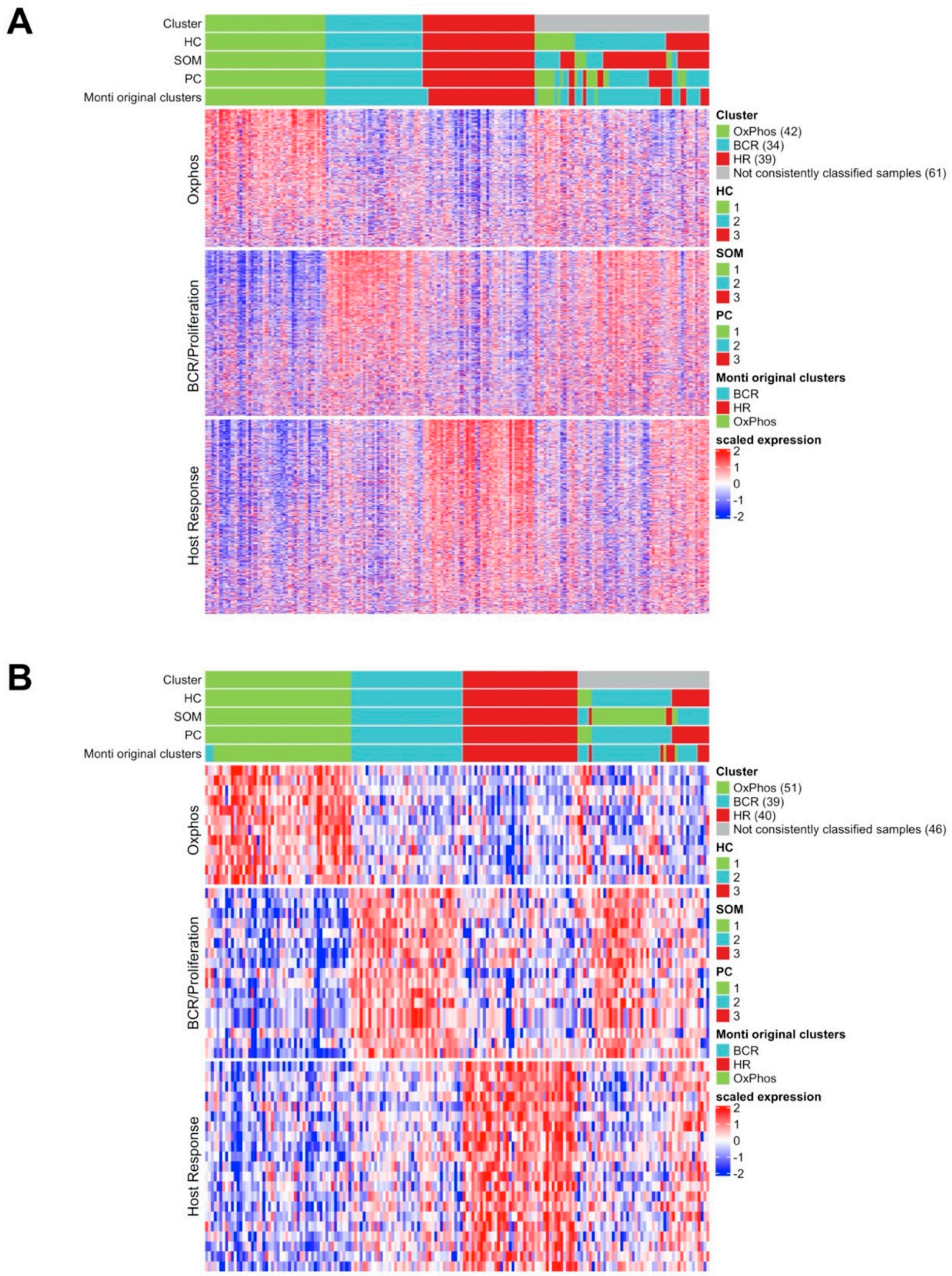

Figure S2. Consensus clusters in original Monti cohort. (**a**) Heatmap indicating the three identified clusters applying our algorithm using all 2118 Monti probes. The upper bars represent the classification of the meta-consensus clusters, hierarchical clustering (HC) only, self-organized maps (SOM) only, probabilistic clustering (PC) only and the original Monti defined clusters, respectively. Two samples were misclassified comparing the meta-consensus clusters to the original Monti classes (**b**) Heatmap indicating the three identified clusters applying our algorithm using the 50 selected probes. The upper bars represent the samples classification of the meta-consensus clusters, hierarchical clustering (HC) only, self-organized maps (SOM) only, probabilistic clustering (PC) only and the original Monti defined clusters, respectively. Three samples were misclassified comparing the meta-consensus clusters to the original Monti classes.

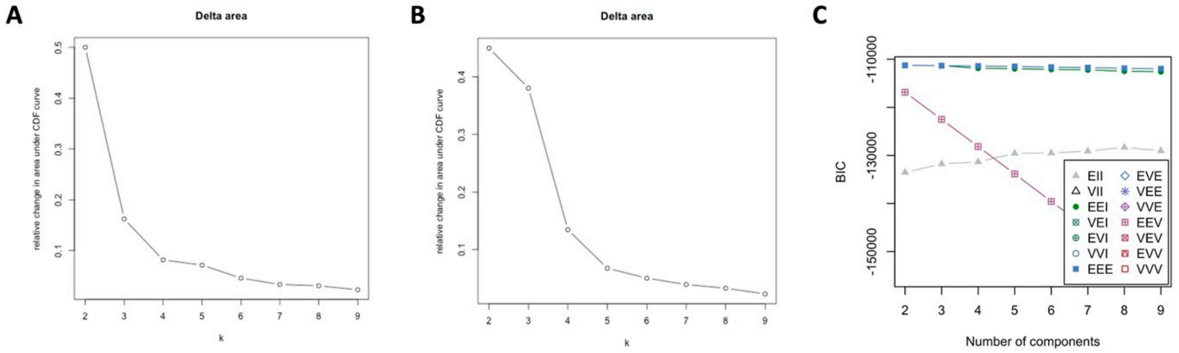

D

	HC - C1	HC – C2	R Total
SOM - C1	43	52	95
SOM - C2	30	50	80
C Total	73	102	175

E

	HC - C1	HC – C2	R Total
PC - C1	95	42	137
PC - C2	0	38	38
C Total	95	80	175

F

	PC - C1	PC – C2	R Total
SOM - C1	59	14	73
SOM- C2	78	24	102
C Total	137	38	175

Figure S3. Identification of consensus clusters in the HOVON-84 cohort using 47 selected genes following the approach as published by Monti. (**A**) Relative change in area under CDF curve for HC algorithm for k from 2 to 9 with 175 samples. (**B**) Relative change in area under CDF curve for SOM algorithm for k from 2 to 9 with 175 samples. (**C**) BIC for PC algorithm for k from 2 to 9 with 175 samples. (**D**) Contingency table between clusters identified by HC and SOM algorithms. (**E**) Contingency table between clusters identified by HC and PC algorithms. (**F**) Contingency table between clusters identified by PC and SOM algorithms. After doing the first step of consensus clustering we tried to re-cluster the non-Host Response subgroup, however the samples didn't differentiate in a new cluster, as in Monti paper.

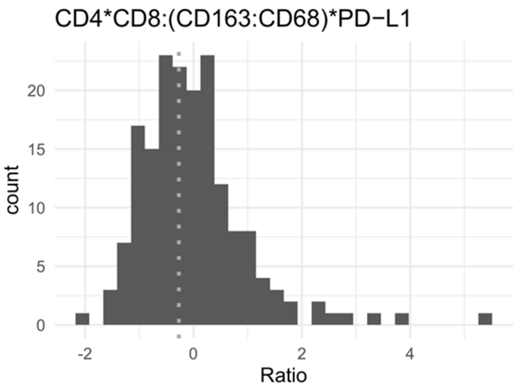

Figure S4. Reproduction of the immune ratio. Distribution of the *CD4***CD8*:(*CD163*:*CD68*)**PD-L1* immuno-ratio for HOVON-84 cohort. The grey line indicates the cut-off (-0.278958829) used to stratify OS in the Keane et al., 2015.

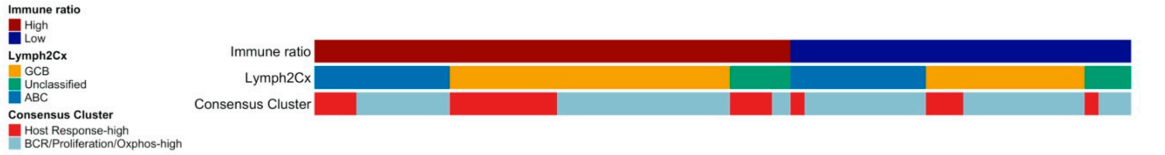

Figure S5. Overlap of Immune ratio, Lymph2CX and Consensus Clusters signatures in the HOVON-84 cohort. There is an association between high Immune ratio and high Host Response. No association with Lymph2Cx was found.

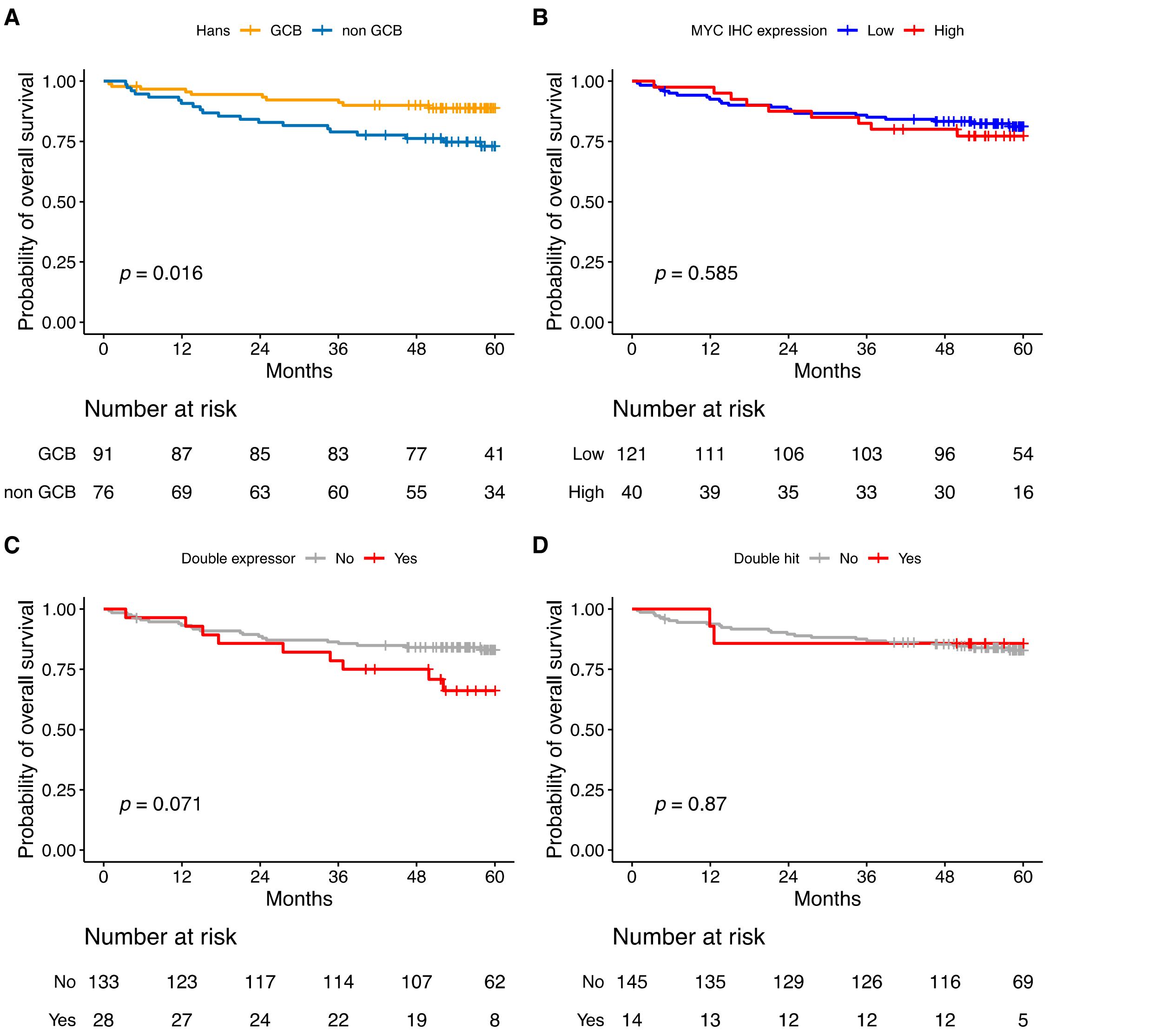

Figure S6. Kaplan Meier curves for overall survival of the HOVON-84 cohort for (**a**) the COO classification defined by Hans. (**b**) the MYC IHC expression low (<50%) and high (>50%) subgroups. (**c**) Double expressor lymphoma. (**d**) Double-hit lymphoma.

Table S1. Overview of characteristics of the HOVON-84 and previously published cohorts.

Characteristic	HOVON-84 (all patients)	HOVON-84 (current study)	Scott et al 2014 [1]	Monti et al 2005 [2]	Carey et al 2015 [3]	Keane et al 2015 [4]
Patients n	574	175	119	176	70	158
Prospective	Yes	Yes	No	Yes	No	Yes
Number of centers	>10	>10	10	1	2	4
Females n (%)	275 (48)	89 (51)	48 (40)	84 (48)	32 (47) – 2 NA	66 (42)
Age≥60 years n (%)	396 (69)	116 (66)		112 (64)	38 (56) – 2 NA	..
Stage II n (%)	114 (20)	40 (23)	53 (46)	53 (32)	..	..
Stage III/IV n (%)	460 (80)	135 (77)	63 (54) – 3 NA	115 (68) – 8 NA	..	..
Extranodal sites >1 n (%)	..	..	13 (12) – 11 NA	21 (12) – 2 NA	..	..
LDH elevated n (%)	379 (66)	104 (59)	53 (55) – 22 NA	81 (56) – 31 NA	..	..
IPI good risk n (%)	246 (43)[aa]	84 (48)[aa]	71 (66)	82 (57)	..	..
IPI poor risk n (%)	328 (57)[aa]	91 (52)[aa]	37 (34) – 11 NA	62 (43) – 32 NA	..	64 (42) – 6 NA
Treatment	R-CHOP	R-CHOP	..	CHOP-based	R-CHOP	R-CHOP
OS events n (%)	164 (29)	37 (21)	..	76 (43)	..	36 (23)
COO ABC n (%)	151 (38)[a]	61 (34)*	49 (41)*	24 (18)[b]	26 (39)[a]	54 (34)[b]
COO GCB n (%)	242 (62)[a] – 181 NA	95 (53)*	48 (40)*	106 (82)[b] – 46 NA	40 (61)[a] – 4 NA	104 (66)[b]

Percentages were calculated under available data. The number of samples with unavailable data are described with NA. [aa] Age adjusted IPI. * COO based on Lymph2Cx algorithm; [a] COO based on Hans algorithm; [b] COO based on Bayesian classifier of 19 genes as previously described by Wright et al. [5]; The treatment is randomized between R-CHOP and RR-CHOP for HOVON-84 cohort with no significant difference between the two groups. NA, not available.

Table S2. List of genes used to classify the four GEP using quantification by the Nanostring platform.

GeneID	Signature	GeneID	Signature	GeneID	Signature
TNFRSF13B	COO	PRMT1	MYC activity	DDX11	Consensus clustering
LIMD1	COO	LDHB	MYC activity	UBA1	Consensus clustering
IRF4	COO	TRAP1	MYC activity	PLCG2	Consensus clustering
CREB3L2	COO	AHCY	MYC activity	CD22	Consensus clustering
PIM2	COO	LRP8	MYC activity	SIPA1L3	Consensus clustering
CYB5R2	COO	EBNA1BP2	MYC activity	CD79A	Consensus clustering
RAB29	COO	CDK4	MYC activity	CD37	Consensus clustering
CCDC50	COO	ETFA	MYC activity	PMS2P9	Consensus clustering
R3HDM1	COO	UCK2	MYC activity	PAX5	Consensus clustering
WDR55	COO	CTPS1	MYC activity	PMS2P2	Consensus clustering
ISY1	COO	GOT2	MYC activity	EZR	Consensus clustering
UBXN4	COO	TMEM97	MYC activity	MAP4K1	Consensus clustering
TRIM56	COO	RRS1	MYC activity	INPP5D	Consensus clustering
MME	COO	DDX21	MYC activity	LAMP1	Consensus clustering
SERPINA9	COO	PHB2	MYC activity	TNFRSF1A	Consensus clustering
ASB13	COO	WDR3	MYC activity	SELPLG	Consensus clustering
MAML3	COO	KIAA0101	MYC activity	CTSB	Consensus clustering
ITPKB	COO	FASN	MYC activity	IFITM1	Consensus clustering
MYBL1	COO	SAMD13	MYC activity	GATA3	Consensus clustering
S1PR2	COO	CDC25A	MYC activity	MAF	Consensus clustering
MYC	MYC activity	LYAR	MYC activity	SLAMF8	Consensus clustering
SRM	MYC activity	SLC12A8	MYC activity	SERPING1	Consensus clustering
AKAP1	MYC activity	P2RY12	MYC activity	TCIRG1	Consensus clustering
NME1	MYC activity	TMEM119	MYC activity	IL6R	Consensus clustering
FBL	MYC activity	SHISA8	MYC activity	CD2	Consensus clustering
RFC3	MYC activity	SLAMF1	MYC activity	TNFSF13	Consensus clustering
TCL1A	MYC activity	COX7A2L	Consensus clustering	CD3E	Consensus clustering
POLD2	MYC activity	PSMA6	Consensus clustering	DAB2	Consensus clustering
RANBP1	MYC activity	RPLP0	Consensus clustering	CD6	Consensus clustering
GEMIN4	MYC activity	MRPL3	Consensus clustering	IRF1	Consensus clustering
MRPS34	MYC activity	NDUFB1	Consensus clustering	MAFB	Consensus clustering
DHX33	MYC activity	ATRAID	Consensus clustering	ITGB2	Consensus clustering
PPRC1	MYC activity	PSMA5	Consensus clustering	CXCL12	Consensus clustering
PPAT	MYC activity	PSMA2	Consensus clustering	GRN	Consensus clustering
FAM216A	MYC activity	SOD1	Consensus clustering	CD4	Immune ratio
PAICS	MYC activity	MRPL15	Consensus clustering	CD8	Immune ratio
UCHL3	MYC activity	DBI	Consensus clustering	CD163	Immune ratio
NOLC1	MYC activity	XRCC5	Consensus clustering	CD68	Immune ratio
RUBCNL	MYC activity	MKI67	Consensus clustering	PDL1	Immune ratio

Table S3. Comparison of cell of origin (COO) allocation between COO classifier and Hans' algorithm.

Lymph2Cx (GEP)	N	IHC Sensitivity	IHC Specificity	IHC PPV	IHC NPV	Hans's Non-GCB (*n* = 76)	Hans's GCB (*n* = 91)	Hans's Not scored (*n* = 8)
**ABC (33%)**	58	91%	84%	78%	94%	51	5	2
**GCB (54%)**	94	84%	84%	94%	78%	14	76	4
**Unclassified (13%)**	23	..	..	..	..	11	10	2

Unclassified and not scored cases were excluded from calculations. ABC, activated B-cell; GCB, germinal center B-cell; GEP, gene expression profiling; IHC, immunohistochemistry; NPV, negative predictive value; PPV, positive predictive value.

Table S4. Performance of MYC activity classifier in the Carey training and HOVON-84 test sets.

Metric	Carey training dataset (30 samples)	HOVON-84 dataset (161 samples)
**Accuracy**	1	0.65
**Sensitivity***	1	0.65
**Specificity**	1	0.65
**PPV**	1 (30/30)	0.43 (105/161)
**NPV**	1 (0/30)	0.82 (56/161)
**Spearman's correlation**	0.96	0.48

Only cases with matched MYC IHC and MYC activity scores were included. The total number of samples equally and not equally classified in comparison to MYC IHC expression are in parenthesis. *The sensitivity refers to the ability of the test to identify tumors with high MYC IHC expression (>50%) as having MYC activity score >0.5.

References:

1. Scott, D.W.; Wright, G.W.; Williams, P.M.; Lih, C.J.; Walsh, W.; Jaffe, E.S.; Rosenwald, A.; Campo, E.; Chan, W.C.; Connors, J.M.; et al. Determining cell-of-origin subtypes of diffuse large B-cell lymphoma using gene expression in formalin-fixed paraffin-embedded tissue. *Blood* **2014**, *123*, 1214–1217. https://doi.org/10.1182/blood-2013-11-536433.
2. Monti, S.; Savage, K.J.; Kutok, J.L.; Feuerhake, F.; Kurtin, P.; Mihm, M.; Wu, B.; Pasqualucci, L.; Neuberg, D.; Aguiar, R.C.; et al. Molecular profiling of diffuse large B-cell lymphoma identifies robust subtypes including one characterized by host inflammatory response. *Blood* **2005**, *105*, 1851–1861. https://doi.org/10.1182/blood-2004-07-2947.
3. Carey, C.D.; Gusenleitner, D.; Chapuy, B.; Kovach, A.E.; Kluk, M.J.; Sun, H.H.; Crossland, R.E.; Bacon, C.M.; Rand, V.; Dal Cin, P.; et al. Molecular classification of MYC-driven B-cell lymphomas by targeted gene expression profiling of fixed biopsy specimens. *J. Mol. Diagn.* **2015**, *17*, 19–30. https://doi.org/10.1016/j.jmoldx.2014.08.006.
4. Keane, C.; Vari, F.; Hertzberg, M.; Cao, K.A.; Green, M.R.; Han, E.; Seymour, J.F.; Hicks, R.J.; Gill, D.; Crooks, P.; et al. Ratios of T-cell immune effectors and checkpoint molecules as prognostic biomarkers in diffuse large B-cell lymphoma: A population-based study. *Lancet Haematol.* **2015**, *2*, e445–e455. https://doi.org/10.1016/s2352-3026(15)00150-7.
5. Sehn, L.H.; Berry, B.; Chhanabhai, M.; Fitzgerald, C.; Gill, K.; Hoskins, P.; Klasa, R.; Savage, K.J.; Shenkier, T.; Sutherland, J.; et al. The revised International Prognostic Index (R-IPI) is a better predictor of outcome than the standard IPI for patients with diffuse large B-cell lymphoma treated with R-CHOP. Blood 2007, 109, 1857–1861. https://doi.org/10.1182/blood-2006-08-038257.
